# FLoCIC: A Few Lines of Code for Raster Image Compression

**DOI:** 10.3390/e25030533

**Published:** 2023-03-20

**Authors:** Borut Žalik, Damjan Strnad, Štefan Kohek, Ivana Kolingerová, Andrej Nerat, Niko Lukač, Bogdan Lipuš, Mitja Žalik, David Podgorelec

**Affiliations:** 1 Faculty of Electrical Engineering and Computer Science, University of Maribor, Koroška cesta 46, SI-2000 Maribor, Slovenia; 2 Department of Computer Science and Engineering, University of West Bohemia, Technická 8, 306 14 Plzeň, Czech Republic

**Keywords:** computer science, algorithm, predictions, interpolative coding, PNG, JPEG LS, JPEG 2000 lossless

## Abstract

A new approach is proposed for lossless raster image compression employing interpolative coding. A new multifunction prediction scheme is presented first. Then, interpolative coding, which has not been applied frequently for image compression, is explained briefly. Its simplification is introduced in regard to the original approach. It is determined that the JPEG LS predictor reduces the information entropy slightly better than the multi-functional approach. Furthermore, the interpolative coding was moderately more efficient than the most frequently used arithmetic coding. Finally, our compression pipeline is compared against JPEG LS, JPEG 2000 in the lossless mode, and PNG using 24 standard grayscale benchmark images. JPEG LS turned out to be the most efficient, followed by JPEG 2000, while our approach using simplified interpolative coding was moderately better than PNG. The implementation of the proposed encoder is extremely simple and can be performed in less than 60 lines of programming code for the coder and 60 lines for the decoder, which is demonstrated in the given pseudocodes.

## 1. Introduction

Data compression is one of the oldest disciplines in computer science [1]. It is present in many computer applications in a wide variety of domains, where it reduces traffic on information channels and supports data archiving. Many data compression approaches have been developed, and reviews of the most important ones can be found in several books [2,3,4,5,6].

Data compression algorithms can be classified as lossless, near-lossless, or lossy. The latter are domain-specific and consider the characteristics of humans’ senses for vision and hearing [7]. Typically, transformations in the frequency domain [8,9,10,11] are used to identify high-frequency components, which are quantized and eliminated permanently. Complete reconstruction is impossible because of this. Other techniques include, domain-specific triangulation [12] or color reductions [13,14]. Lossy methods cannot guarantee a distortion rate below the chosen limit at the level of an individual element (e.g., a pixel), which is why the near-lossless methods have been developed [15]. They enable users to specify exactly to what extent the errors in the reconstructed data are acceptable. The lossless methods [16] reconstruct the original data exactly. In some domains, they are indispensable, such as compressing text, medical images, or high-quality sound, especially for editing purposes, to prevent accumulation of compression errors through repetitive compression and decompression.

This paper introduces a new approach for lossless compression of continuous-tone raster images (photos). The suitability of interpolative coding for this type of image is examined after experiments with prediction functions. The main contribution of this paper can be summarized as follows:

- An evaluation of a new local pixel prediction model;
- A simplification of interpolative coding;
- Testing the suitability of interpolative coding for continuous-tone image compression;
- Comparison of the proposed data compression approach with PNG, JPEG LS, and JPEG 2000 in lossless mode;
- A compact programming code.

This paper consists of five sections. Section 2 explains briefly the backgrounds of JPEG LS, PNG, and JPEG 2000 in lossless mode. Section 3 introduces the proposed Few Lines of Code raster Image Compressor (FLoCIC) method. The corresponding pseudocodes are given in this Section. An evaluation of the method is given in Section 4. Section 5 concludes the paper.

## 2. Background

**Figure 1:**
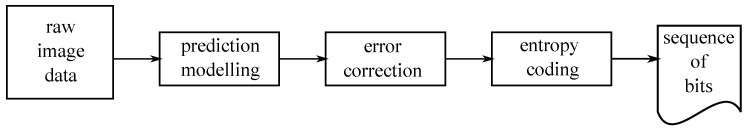
Typical components of the lossless image compression pipeline.

Let $P=\langle p_{x,y}\rangle$, 0≤x<X, 0≤y<Y be a *t* bit plane, continuous-tone grayscale raster image (t>1) with a resolution of X×Y pixels, where $p_{x,y}\in \left[0,1,\ldots ,2^{t}-1\right]$. The lossless image compression methods follow the idea shown schematically in Figure 1.

P should be processed in a predefined order, commonly in the raster-scan way. The value of the processed pixel $p_{x,y}\in P$ is estimated first by the prediction function $f(L_{x,y})$, which uses the values of some already-processed pixels, where $L_{x,y}=\left\{p_{i,j}\right\}$, j<y or j=yandi<x. Prediction models where *L* consists of just the neighboring pixels in the close proximity of $p_{x,y}$ will be considered local predictors.

The predicted value is then subtracted from the value of the processed pixel (see Equation (1)), and a prediction error $\epsilon_{x,y}$ is obtained:

(1) $$\epsilon_{x,y}=p_{x,y}-f\left(L_{x,y}\right).$$

Although the domain of $\epsilon_{x,y}\in \left[-2^{t}+1,2^{t}-1\right]$ is larger than the domain of $p_{x,y}\in P$, its information entropy is expected to be smaller. Namely, the conventional distribution of the $\epsilon_{x,y}$ values follows the geometric distribution [17], which offers a good opportunity for information entropy reduction [4].

The prediction values can be corrected further in the second step of the compression pipeline (see Figure 1) using context-based models [17,18,19]. Many methods, however, omit this step and proceed directly with the encoding, where RLE, Huffman, arithmetic, or dictionary-based encoding is used (or a combination of them) [16].

A very brief overview of JPEG LS and JPEG 2000 in lossless mode and PNG (the formats used for the comparison in Section 4) is given in the continuation.

Joint Photographic Experts Group—Lossless (JPEG LS): After the success of the JPEG standard, the same group of experts continued the work on lossless (and the near-lossless) image compression. JPEG LS was published in 1999 (ISO/IEC 14495-1), and the extensions followed four years later (ISO/IEC 14495-2) [20]. JPEG LS consists of a regular and RLE mode. Only the regular mode is considered briefly for the purposes of this paper.

**Figure 2:**
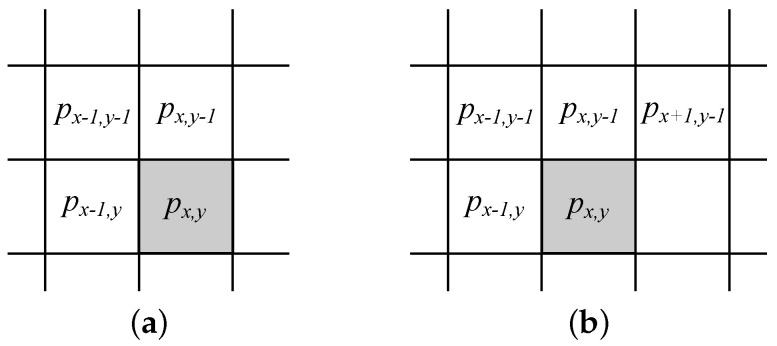
Pixels used in (**a**) JPEG LS prediction and (**b**) for context modeling.

JPEG LS follows the ideas developed in LOCO-I [17] and includes all steps from Figure 1. $L_{x,y}$ contains three neighboring pixels as shown in Figure 2a (i.e., $L_{x,y}=\{p_{x-1,y}$, $p_{x-1,y-1},p_{x,y-1}\}$). The prediction function $f(L_{x,y})$ is shown in Equation (2).

(2) $$f_{x,y}=\left\{\begin{array}{cc} \min(p_{x-1,y},p_{x,y-1}); & \mathrm{when}p_{x-1,y-1}\geq \max\left(p_{x-1,y},p_{x,y-1}\right)\\ \max(p_{x-1,y},p_{x,y-1}); & \mathrm{when}p_{x-1,y-1}\leq \min\left(p_{x-1,y},p_{x,y-1}\right)\\ p_{x-1,y}+p_{x,y-1}-p_{x-1,y-1}; & \mathrm{otherwise}. \end{array}\right.$$

A mechanism for the prediction correction is used after $\epsilon_{x,y}$ is determined. For this, three gradients for $\Delta_{i}$, where i∈{1,2,3}, are calculated using Equation (3) (see Figure 2b):

(3) $$\begin{array}{c} \Delta_{1}=p_{x+1,y-1}-p_{x,y-1}\\ \Delta_{2}=p_{x,y-1}-p_{x-1,y-1}\\ \Delta_{3}=p_{x-1,y-1}-p_{x-1,y} \end{array}$$

As the number of all possible combinations of the three gradient values for an image with t=8 is a huge $511^{3}$, it is brought down by a reduction function to a manageable 355 values, which represent the entry points into the context models. These models improve adaptively during the image compression process and serve for correcting $\epsilon_{x,y}$. Details can be found in [5,17].

The corrected values $\epsilon_{x,y}$ are encoded with Golomb codes [21]. Golomb’s parameter is also obtained from the context model. However, as this coding lacked efficiency, the arithmetic coding was added to the standard in 2003. In this way, JPEG LS became the best lossless compression standard which uses only the local predictors. Unfortunately, its usage was limited due to patents. This is why the PNG standard has become the most popular format for lossless raster image compression.

Portable Network Graphics (PNG): This was designed as a replacement for the GIF format, which contained the patent-protected LZW [22] compression algorithm. The development started as an open project of many individuals [23]. PNG was soon accepted by the W3C consortium, which boosted its popularity. In 2004, it became an international standard (ISO/IEC 15948).

PNG performs the prediction on the level of a raster scan line. It applies five predictors (named filters), where $L_{x,y}$ is defined as follows:

**None:**    $L_{x,y}=\emptyset$;
**Sub:**    $L_{x,y}=\left\{p_{x-1,y}\right\}$;
**Up:**           $L_{x,y}=\{p_{x,y-1}$};
**Average:**     $L_{x,y}=\left\{p_{x,y-1},p_{x-1,y}\right\}$;
**Paeth:**    $L_{x,y}=\left\{p_{x,y-1},p_{x-1,y},p_{x-1,y-1}\right\}$.

The filter average calculates the average values of two pixels in $L_{x,y}$, while the Paeth filter is determined by the algorithm given in [24]. The best predictor is then applied on the whole line. PNG does not use any context-based corrections for $\epsilon_{x,y}$. The open-source algorithm Deflate [5] is used in the final step. It is based on the LZ77 algorithm [25], whose tokens are then compressed by Huffman coding [26]. PNG is still the most popular lossless image compression format.

JPEG 2000 in lossless mode: JPEG 2000 is another standard from the JPEG consortium whose primary goal was to achieve excellent lossy compression with support for scalability [27]. It is based on the wavelet transform. The Le Gall–Tabatabai wavelet [28] was used for lossless compression as it operates with integer coefficients only. JPEG 2000 does not perform any prediction nor any correction of the predicted error. Instead, it explores the properties of the hierarchical wavelet transform to compress the obtained coefficients efficiently with the specially designed arithmetic encoder, namely with MQ-coder [29].

There are, however, other prediction models. An overview of them can be found in a very recent paper by Ulacha and Łazoryszczak [30].

## 3. Materials and Methods

The new prediction model, used later in experiments, is introduced first. An explanation of interpolative encoding and its simplifications is given after that.

### 3.1. Multifunction Local Predictions

A new prediction mechanism was tried, although the prediction suggested in JPEG LS (Equation (2)) has been proven to work well. Let us have a set of predictors $F_{x,y}=\left\{f_{i}\left(L_{x,y}\right)\right\},0\leq i<I$, where *I* is the number of functions $f_{i}$ and $L_{x,y}$ is a set of some already-seen neighboring pixels. Function MinF, given by Equation (4), returns the index *i* of $f_{i}\left(L_{x,y}\right)$, which achieves the minimal prediction error:

(4) $$MinF\left(F_{x,y}\right)=argmin_{i}\left\{\right|p_{x,y}-f_{i}\left(L_{x,y}\right)\left|\right\}$$

We supposed that if the $i^{th}$ predictor achieved the smallest $|\epsilon_{x,y}|$ for $p_{x,y}$, then most of the time, the same predictor was also the best one for the neighboring pixel, (i.e., for the next right $p_{x+1,y}$ or for the next bottom pixel $p_{x,y+1}$).

Table 1 shows a set of the predictors used in our case, when $L_{x,y}=\{p_{x-1,y},$$p_{x-1,y-1},$$p_{x,y-1},$$p_{x+1,y-1}\}$ and I=12. The first pixel $p_{0,0}$ cannot be predicted, while the function $f_{0}$ is applied only for the remaining pixels $p_{x,0}$ (i.e., for the pixels in the first row of P). Similarly, the function $f_{1}$ is used for pixels $p_{0,y}$.

### 3.2. Interpolative Coding

The idea of interpolative coding (IC), proposed by Moffat and Stuiver in 2000 [31], differs drastically from other compression methods. For example, the statistically based approaches, such as Huffman or arithmetic coding, assign a unique prefix code to each symbol of the message [5]. Dictionary-based approaches (i.e., LZ family compression algorithms) construct phrases from the messages and assign them unique tokens [6]. The symbols from the input message are processed in the given sequence in both cases. On the other hand, IC processes the input message in an arbitrary yet predefined way, where the code of a particular symbol depends more on its position than on its value.

The message was, in our case, obtained from the prediction step of the compression pipeline (see Figure 1); in other words, it is sequence is $E=\langle \epsilon_{i}\rangle$, 0≤i<n, $\epsilon_{i}\in \left\{-2^{t}+1,2^{t}-1\right\}$, where *t* is the bit plane depth (see Section 2). The raster scan traversal transforms (x,y)→i=y·X+x, and therefore n=X·Y.

IC works in two steps: initialization and encoding.

**Initialization:**  E is transformed first into a sequence of non-negative integers $N=\langle \epsilon_{i}^{+}\rangle$, $\epsilon_{i}^{+}\in \left\{0,2^{t+1}-1\right\}$, 0≤i<n by Equation (5), which interleaves the input positive and negative values:

(5) $$\epsilon_{i}^{+}=\left\{\begin{array}{cc} \epsilon_{i}; & \mathrm{when}\;i=0,\\ 2\epsilon_{i}; & \mathrm{when}\;i>0\;\mathrm{and}\;\epsilon_{i}\geq 0,\\ 2|\epsilon_{i}|-1; & \mathrm{when}\;i>0\;\mathrm{and}\;\epsilon_{i}<0. \end{array}\right.$$

N is then used to obtain a strictly increasing cumulative sequence $C=\langle c_{i}\rangle$, 0≤i<n with Equation (6):

(6) $$c_{i}=\left\{\begin{array}{cc} \epsilon_{0}^{+}; & \mathrm{when}i=0,\\ 1+\epsilon_{i}^{+}+c_{i-1}; & \mathrm{when}0<i<n. \end{array}\right.$$

**Encoding:** The original IC mechanism, as described in [31], is given first, and our modification, which simplifies the encoding process, is explained after that. IC works through a recursive dividing of C in half according to Equation (7), where *L* denotes the low guard and *H* is the high guard of the considered part of C:

(7) $$\begin{array}{c} m=\left\lfloor \frac{L+H}{2}\right\rfloor \end{array}$$

Then, $c_{m}$ is encoded in three steps:

1. A range $G=[g_{L},g_{H}]$ of all possible values is determined first (see Equation (8)) by taking into account that C is strictly monotone:
  (8) $$\begin{array}{c} \begin{array}{c} g_{L}=c_{L}+\left(m-L\right),\\ g_{H}=c_{H}-\left(H-m\right). \end{array} \end{array}$$
2. The number of bits *g* needed to encode all possible values from *G* is then calculated with Equation (9):
  (9) $$\begin{array}{c} g=\lceil \log_{2}\left(g_{H}-g_{L}+1\right)\rceil . \end{array}$$
3. Finally, the value $v=c_{m}-g_{L}$ is encoded in binary with *g* bits and sent to the output $B=\langle b_{i}\rangle$, where $b_{i}\in \left\{0,1\right\}$ and 0≤i<|B| are bits and |B| is the total number of bits.

IC also has a special (i.e., the best) case, which may increase its efficiency drastically. When $H-L=c_{H}-c_{L}$, IC does not need to send any bits at all to B. In particular, this case is trivially detectable by a decoder. The interval between *L* and *H* is filled simply by incrementing the value $g_{L}$. A similar case was recognized in [32,33]. If $H-L=D\cdot (c_{H}-c_{L}$), where *D* is the maximal value of the domain, then the encoder also does not emit any bits. However, this case is extremely rare in image compression after applying a prediction. Therefore, it is not worth using it in this application.

The encoding in step 3 can be completed in different ways: classical binary codes, truncated binary code [5], FELICS codes [34], and Ψ codes (in the case of a small alphabet), as suggested in [32].

**Simplifying the interpolative encoding process:** IC, as described above and proposed in [31], can be simplified further. Specifically, if the requirement of a strictly increasing cumulative sequence of integers is released, then the whole procedure becomes simpler as follows:

- C is obtained from N with Equation (10):
  (10) $$c_{i}=\left\{\begin{array}{cc} \epsilon_{0}^{+}; & \mathrm{when}i=0,\\ \epsilon_{i}^{+}+c_{i-1}; & \mathrm{otherwise}. \end{array}\right.$$
- Calculation of guards $g_{L}$ and $g_{H}$ is not needed, as the range containing the value $c_{m}$ is simply $G=[c_{L},c_{H}]$.
- Detection of the optimal case is simplified to check whether $c_{H}=c_{L}$.

Finally, it should be noted that the simplified version does not shorten B. Indeed, the original and simplified versions of IC generate the same stream of bits.

In [35] it was reported that IC can be a good alternative to arithmetic coding for bi-level image compression. IC was also successful at chain code compression [32,33,36]. The characteristic of both domains is a small alphabet. However, to the best of our knowledge, IC has not been used for compression of continuous-tone images, as is the case in this application.

#### 3.2.1. An Example

**Figure 3:**
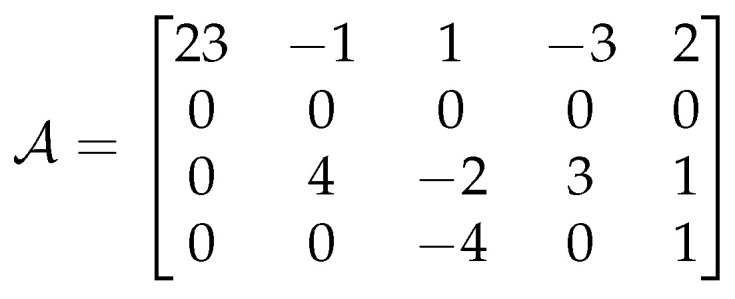
Example: The matrix contains the values of ϵ after the prediction process.

**Figure 4:**
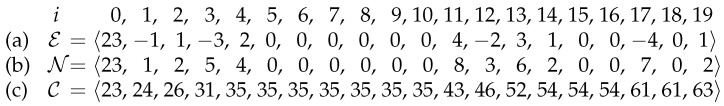
Example: (**a**) A sequence of prediction errors. (**b**) A sequence of interleaved values. (**c**) The running sum sequence of cumulative integer values.

A short example is given to clarify the encoding process. Let us suppose that a prediction model has a generated matrix A containing error values $\epsilon_{x,y}$ (see Figure 3). The first element in the matrix represents the absolute pixel value which, of course, cannot be predicted. A is then used to obtain E, shown in Figure 4a, with the raster scan traversal. Applying Equation (5) yields N (see Figure 4b), from which C is obtained with Equation (10) (see Figure 4c). The indices of all the sequences are shown at the top of Figure 4.

**Figure 5:**
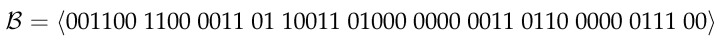
Example: Result of encoding.

The simplified interpolative coding initializes L=0 and H=19. The interval $G=[c_{L}=23,c_{H}=63]$ is set, and m=9 is calculated using Equation (7). The number of bits $g=\lceil \log_{2}\left(63-23+1\right)\rceil =6$ for encoding all possible values from *G*. The value *v* is then calculated as $v=c_{m}-c_{L}=35-23=12$ and binary encoded with g=6 bits. The algorithm now proceeds recursively as demonstrated in Table 2, while the resulting sequence B is given in Figure 5. Binary encoding was used in this example for *v* due to clarity. Applying truncated binary codes or FELICS codes would yield a shorter B.

Finally, the pseudocodes are given for FLoCIC: Algorithm 1 performs the initialization, Algorithm 2 implements the JPEG LS prediction, and Algorithm 3 presents the interpolative coder.
**Algorithm 1** Lossless image compression with FLoCIC1:**function**  CompressWithFLoCIC(P, *X*, *Y*)      ▹ returns binary sequence B2:                   ▹P: raw image; X,Y: image resolution3:  E← Predict(P, *X*, *Y*)4:  n←X×Y5:   $N_{0}\leftarrow E_{0}$6:   **for** **i**← 1, n−1**do**           ▹ turns $\epsilon_{i}\in E$ to non-negative values7:    **if** $\epsilon_{i}\geq 0$ **then**8:     $N_{i}\leftarrow 2\times \epsilon_{i}$9:    **else**10:     $N_{i}\leftarrow 2\times \mathrm{abs}\left(\epsilon_{i}\right)-1$11:    **end if**12:   **end for**13:   $C_{0}\leftarrow N_{0}$14:   **for** **i**← 1, n−1 **do**             ▹ forms the cumulative sequence15:    $C_{i}\leftarrow C_{i-1}+N_{i}$16:   **end for**17:   $B\leftarrow \mathrm{SetHeader}(X,c_{0},c_{n-1},n)$18:   B←IC(B,C,0,n−1)       ▹ call simplified interpolative coding19:   **return** B20:**end function**

**Algorithm 2** JPEG LS prediction
1:**function**  Predict(P, *X*, *Y*)            ▹ return a sequence of predicted values2:    **for** x←0,X−1**do**             ▹P: raw image; X,Y: image resolution3:    **for** y←0,Y−1 **do**4:      **if** x=0 **and** y=0 **then**            ▹ first element is not predicted5:       $E_{y*X+x}\leftarrow p_{0,0}$6:      **else if** y=0 **then**                        ▹ first row7:       $E_{y*X+x}\leftarrow p_{x-1,0}-p_{x,0}$8:      **else if** x=0 **then**                       ▹ left column9:       $E_{y*X+x}\leftarrow p_{0,y-1}-p_{0,y}$10:      **else if** $p_{x-1,y-1}\geq \max\left(p_{x-1,y},p_{x,y-1}\right)$ **then**     ▹ for all remaining rows11:       $E_{y*X+x}\leftarrow$**min**$\left(p_{x-1,y},p_{x,y-1}\right)-p_{x,y}$12:      **else if** $p_{x-1,y-1}\leq \max\left(p_{x-1,y},p_{x,y-1}\right)-p_{x,y}$ **then**13:       $E_{y*X+x}\leftarrow$ max$\left(p_{x-1,y},p_{x,y-1}\right)-p_{x,y}$14:      **else**15:       $E_{y*X+x}\leftarrow p_{x-1,y}+p_{x,y-1}-p_{x-1,y-1}-p_{x,y}$16:      **end if**17:     **end for**18:   **end for**19:   **return** E20:
**end function**



**Algorithm 3** Simplified interpolative coding
1:**function**  IC(B,C,L,H)   ▹B: sequence of bits; C: cumulative sequence; L,H: guards2:   **if** H−L>1 **then**3:    **if** $c_{H}\neq c_{L}$ **then**4:     m←⌊0.5×(H+L)⌋          ▹ position of the coded element5:     $g\leftarrow \lceil \log_{2}\left(c_{H}-c_{L}+1\right)\rceil$          ▹ number of needed bits6:     $B\leftarrow \mathrm{Encode}(B,g,c_{m}-c_{L})$   ▹ insert ordinary, truncated, or FELICS codes7:     **if** L<m **then**8:      IC(B,C,L,m)9:     **end if**10:     **if** m<R **then**11:      IC(B,C,m,R)12:     **end if**13:    **end if**14:   **end if**15:
**end function**



#### 3.2.2. Decoding

The decoder needs the following data to restore C:

- The values of the first $c_{0}$ and the last element $c_{n-1}$;
- The length *n*;
- The sequence of bits B.

**Figure 6:**
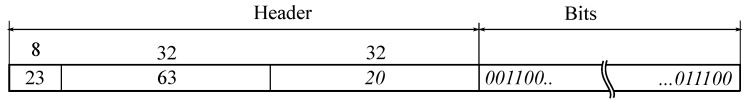
Example: Storing the results of interpolative coding.

The first three items form the header, while B is stored after it. In our case, 8 bits were reserved for $c_{0}$, while for $c_{n-1}$ and *n*, 32 bits were allocated (i.e., the header occupied 72 bits in total). The content of the header for the example from Section 3.2.1 is in Figure 6 and given in decimals. When coding raster images, its resolution in the *X* direction should be added to the header, as *Y* can be obtained by Y=n/X.

**Figure 7:**
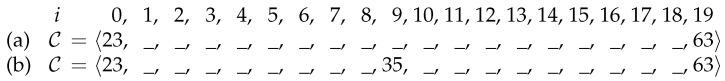
Example of decoding. (**a**) Situation after initialization. (**b**) Decoded element at m=9.

Decoding starts with reading the header, allocating the n=20 memory units for sequence C, and initializing $c_{0}=23$ and $c_{n-1}=63$ (see Figure 7a). The decoder sets L=0 and H=n−1=19. As $c_{0}\neq c_{19}$, m=9 is calculated with Equation (7). The number of bits *g*, which were used for encoding $c_{9}$, is then calculated as $g=\lceil \log_{2}\left(63-23+1\right)\rceil =6$. Therefore, the first 6 bits are read from B (i.e., bits 001100, corresponding to v=12). Finally, $c_{L}+v$ reconstructs 35, which is written at $c_{9}$ (see Figure 7b). The decoder now operates recursively, mirroring the coding process. If $c_{L}=c_{H}$, then the decoder sets $c_{i}=c_{L}$, L<i<H and does not read any bit from B. Algorithm 4 shows the FLoCIC decoder, while Algorithms 5 and 6 contain the inverse simplified interpolative decoder and the inverse JPEG LS predictor, respectively. From all the pseudocodes, it is evident that the FLoCIC’s coder and decoder were completely symmetrical. FLoCIC’s programming code is accessible in [37].
**Algorithm 4** Image decompression with FLoCIC1:**function**  DecompressWithFLoCIC(B)            ▹B: sequence of bits2:                 ▹ Function returns reconstructed raw image P3:  $\mathrm{DecodeHeader}(B,X,n,c_{0},c_{n-1})$4:  Y←n/X5:  $C\leftarrow \mathrm{InitialiseC}(n,c_{0},c_{n-1})$  ▹ Create C with *n* elements and set $C_{0}$ and $C_{n-1}$6:  C←DeIC(B,C,0,n−1)     ▹ Reconstruct remaining elements of C7:  $N_{0}\leftarrow C_{0}$8:  **for** i←1,n−1 **do**        ▹ Calculate non-cumulative sequence N9:   $N_{i}\leftarrow C_{i}-C_{i-1}$10:  **end for**11:  $E_{0}\leftarrow N_{0}$12:  **for** i←1,n−1 **do**   ▹ Unwrap the values to get the errors in the prediction13:   **if** $\mathrm{Even}(N_{i}$) **then**14:    $E_{i}\leftarrow N_{i}/2$15:   **else**16:    $E_{i}\leftarrow -\left(N_{i}+1\right)/2$17:   **end if**18:  **end for**19:  P=PredictInverse(E,X,Y)20:  **return** P21:**end function**

**Algorithm 5** Simplified interpolative decoding
1:**function**  DeIC(B,C,L,H)           ▹B: sequence of bits to be decoded2:        ▹C: sequence to be reconstructed after all recursive calls are executed3:                                ▹L,H: guards4:   **if** $c_{L}=c_{H}$ **then**                ▹ cheching for the special case5:    **for** i←L+1,H−1 **do**6:     $C_{i}\leftarrow c_{L}$7:    **end for**8:   **else**9:    m←⌊0.5×(H+L)⌋       ▹ position of the element to be decoded10:    $g\leftarrow \lceil \log_{2}\left(c_{H}-c_{L}+1\right)\rceil$             ▹ get number of bits11:    B←GetBits(B,g)                  ▹ read *g* bits from B12:    $C_{m}\leftarrow \mathrm{Decode}\left(B\right)$  ▹ Decode ordirani, trunacated, or FELICS binary code13:    **if** L<m **then**      ▹ proceed recursively with the reconstruction of C14:     DeIC(B,C,L,m)15:    **end if**16:    **if** m<H **then**17:     DeIC(B,C,m,H)18:    **end if**19:    **end if**20:
**end function**



**Algorithm 6** Inverted JPEG LS predictor
1:**function**  PredictInverse(E, *X*, *Y*)    ▹ return reconstructed raw image data in P2:  **for** x←0,X−1 **do**  ▹E: sequence of prediction errors; X,Y: image resolution3:   **for** y←0,Y−1 **do**4:    **if** x=0 **and** y=0 **then**           ▹ first element is not predicted5:     $p_{0,0}\leftarrow E_{0}$6:     **else if** y=0 **then**                      ▹ first row7:      $p_{x,0}\leftarrow p_{x-1,0}+E_{y*X+x}$8:    **else if** x=0 **then**                     ▹ left column9:     $p_{0,y}\leftarrow p_{0,y-1}+E_{y*X+x}$10:    **else if** $p_{x-1,y-1}\geq \max\left(p_{x-1,y},p_{x,y-1}\right)$ **then**   ▹ for all remaining rows11:     $p_{x,y}\leftarrow$ min $\left(p_{x-1,y},p_{x,y-1}\right)+E_{y*X+x}$
12:    **else if** $p_{x-1,y-1}\leq \max\left(p_{x-1,y},p_{x,y-1}\right)$ **then**13:     $p_{x,y}\leftarrow$ max $\left(p_{x-1,y},p_{x,y-1}\right)+E_{y*X+x}$14:    **else**15:     $p_{x,y}\leftarrow p_{x-1,y}+p_{x,y-1}-p_{x-1,y-1}+E_{y*X+x}$16:    **end if**17:   **end for**18:  **end for**19:  **return** P20:
**end function**



## 4. Experiments

**Figure 8:**
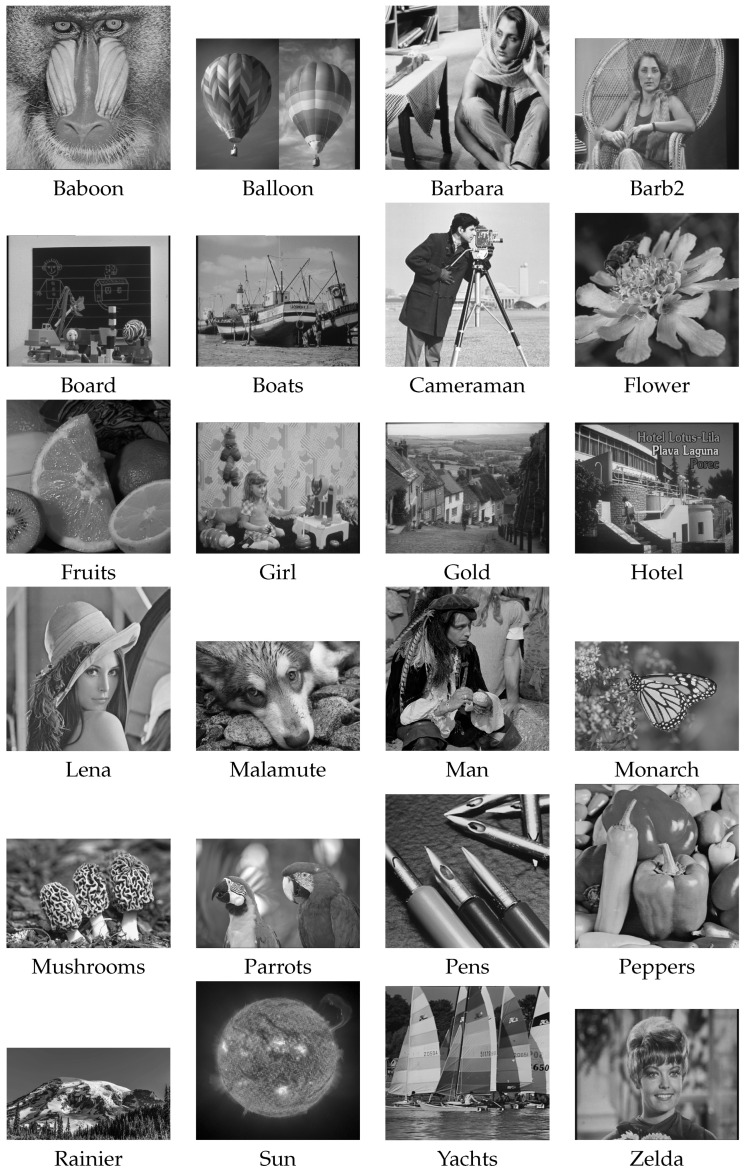
Testing raster images.

Twenty-four popular benchmark grayscale images with t=8 were used in the experiments (see Figure 8). Table 3 introduces in the first three columns the information about these images, including their resolutions, raw sizes in bytes, and the values of the raw data information entropy ($H_{raw}$) [4]. The remaining three columns show the effect of the information entropy reduction after applying three different predictors: the first two are the multifunction predictors (see Section 3.1), ($H_{NR}$ is the information entropy when the next right pixel is predicted, and $H_{NB}$ stands for the next bottom pixel.) while $H_{JPEGLS}$ is the predictor used in JPEG LS (see Equation (2)). The last column contains the average absolute prediction error $\bar{E}$ obtained when the JPEG LS predictor was used. Although the differences between the obtained information entropies were small, it can be concluded that the JPEG LS predictor is better. Indeed, in all cases except for the Peppers image, it reduced the information entropy the best. The JPEG LS predictor was therefore used in the continuation.

FLoCIC was compared against JPEG LS, JPEG 2000 in lossless mode, and PNG. The results are given in Table 4. The JPEG LS images were generated by IrfanView’s JPEG LS plug-in [38], while the JPEG 2000 in lossless mode and PNG images were obtained by ImageMagick [39]. FLoCIC was, of course, coded by ourselves. Our implementation of the arithmetic coding (AC) based on the E1, E2 and E3 transforms [40] was used to confront it with IC.

JPEG LS performed the best, and JPEG 2000 in lossless mode was second. FLoCIC outperformed PNG slightly, either when IC or AC was used in the final step. Surprisingly, IC combined with the FELICS codes [34] turned out to be moderately better than AC on average. However, it should be stressed that the most basic implementation of AC was used. For example, context-based adaptive binary arithmetic coding [41] would yield better results.

As can be seen, FLoCIC worked successfully with images of different resolutions. Just for the reader’s information, the largest image, *Sun*, was compressed in 0.793 s, while the more than 64 times smaller image, *Cameraman*, was compressed in 0.018 s on a very modest computer: an Intel i5-2500K processor with 3.3 GHz with 16 GB of RAM running Windows 10. FLoCIC was implemented in C++ and compiled with Visual Studio 19. Decompression was approximately 15% faster, as decoding the FELICS codes was faster than encoding them.

At this point, it should be stressed that none of these methods are competitive with the modern lossless image compression approaches, such as JPEG XL [42] or WebP [43] in lossless mode. They do not perform a local prediction but instead investigate larger areas of pixels.

## 5. Discussion

This paper introduces a new, very simple algorithm for lossless image compression named few lines of code raster image compression (FLoCIC). Indeed, as shown in the given pseudocode, less than 60 lines of programming code are needed for it. The code is, however, even shorter when coded in, for example, C++. The compression pipeline is classical, consisting of only two parts: the prediction (the JPEG LS predictor turned out to be the most successful) and the entropy encoder. Interpolative coding, a technique developed by Moffat and Stuiver [31], is less known and has not been used in image compression, except for bi-level images [32,33,35]. It turned out to be as good as the widely used arithmetic coding for images with continuous tones as well. In this paper, we simplified interpolative coding, leading to further shortening of the programming code.

Twenty-four classical benchmark 8 bit grayscale images were used to evaluate the effectiveness of FLoCIC. They had different resolutions, ranging from 256×256 up to 2100×2034 pixels. Concerning the compression ratio achieved, FLoCIC can cope with PNG, the most widely used lossless image compression standard. In the given set of testing images, FLoCIC turned to actually be slightly better and moderately worse than JPEG 2000. JPEG LS was, however, better by almost 10 %. It is the only one of the considered approaches that incorporates the correction of prediction errors. Despite being efficient, JPEG LS is rarely found in practice. It should be noted, however, that none of the mentioned approaches are competitive according to the compression ratio with the state-of-the-art JPEG XL or WebP. However, they do not use the simple and fast local prediction techniques and instead employ the wider pixel’s surroundings.

FLoCIC is an interesting alternative to PNG. It is extremely easy to implement, and as such, it could be applied in an environment with modest computational power, such as in embedded systems [44]. It is also suitable for programming training for students of computer science, similar to, for example, Delaunay triangulation [45,46].

## Figures and Tables

**Table 1:** Set of predictors F.

$f_{0}=p_{x-1,y}$	$f_{6}=\left\lfloor 0.5\cdot \left(p_{x-1,y-1}+p_{x,y-1}\right)\right\rfloor$
$f_{1}=p_{x,y-1}$	$f_{7}=\left\lfloor 0.5\cdot \left(p_{x,y-1}+p_{x+1,y-1}\right)\right\rfloor$
$f_{2}=p_{x-1,y-1}$	$f_{8}=\left\lfloor 0.5\cdot \left(p_{x-1,y}+p_{x+1,y-1}\right)\right\rfloor$
$f_{3}=p_{x+1,y-1}$	$f_{9}=\left\lfloor 0.5\cdot \left(p_{x-1,y-1}+p_{x+1,y-1}\right)\right\rfloor$
$f_{4}=p_{x-1,y}+p_{x,y-1}-p_{x-1,y-1}$	$f_{10}=p_{x,y-1}+p_{x-1,y-1}-p_{x+1,y-1}$
$f_{5}=\left\lfloor 0.5\cdot \left(p_{x-1,y}+p_{x-1,y-1}\right)\right\rfloor$	$f_{11}=p_{x,y-1}+p_{x-1,y-1}-p_{x-1,y}$

**Table 2:** An example of simplified interpolative coding.

*L*	*H*	*m*	$c_{m}$	$G=[c_{L},c_{H}]$ ^1^	*g*	*v* ^2^	Code
0	19	9	35	[23, 63]	6	12	001100
0	9	4	35	[23, 35]	4	12	1100
0	4	2	26	[23, 35]	4	3	0011
0	2	1	24	[23, 26]	2	1	01
4	9	/	/	[35, 35]	/	/	/ ^3^
9	19	14	54	[35, 63]	5	19	10011
9	14	11	43	[35, 54]	5	8	01000
9	11	10	35	[35, 43]	4	0	0000
11	14	12	46	[43, 54]	4	3	0011
12	14	13	52	[46, 54]	4	6	0110
14	19	16	54	[54, 63]	4	0	0000
14	16	/	/	[54, 54]	/	/	/ ^3^
16	19	17	61	[54, 63]	4	7	0111
17	19	18	61	[61, 63]	2	0	00

^1^ The calculation of the interval’s guards according to Equation (8) is not needed in the simplified version. ^2^ Remember that $v=c_{m}-c_{L}$. ^3^ As $C_{L}=C_{H}$, the coder does not output any bits.

**Table 3:** Information about the images’ resolutions and raw sizes in bytes, entropy of the raw images, entropies for three prediction models, and average absolute prediction errors for JPEG LS predictor.

Image	Resolution	Raw Size	$H_{\mathrm{raw}}$ ^1^	$H_{\mathrm{NR}}$ ^2^	$H_{\mathrm{NB}}$ ^3^	$H_{\mathrm{JPEGLS}}$ ^4^	$\bar{E}$
Baboon	512×512	262,144	7.357	6.499	6.414	6.275	14.342
Balloon	720×576	414,720	7.346	3.282	3.204	3.120	1.608
Barbara	512×512	262,144	7.343	5.794	5.890	5.758	12.031
Barb2	720×576	414,720	7.484	5.490	5.238	5.181	6.895
Board	720×576	414,720	6.828	4.073	4.013	3.947	2.927
Boats	720×576	414,720	7.088	4.527	4.394	4.307	3.707
Cameraman	256×256	65,536	6.904	5.200	5.273	5.150	8.214
Flower	512×480	245,760	7.410	3.881	3.889	3.866	2.755
Fruits	512×480	245,760	7.366	4.173	4.170	4.014	3.062
Girl	720×576	414,720	7.288	4.354	4.153	4.207	3.391
Gold	720×576	414,720	7.530	4.959	4.951	4.716	4.716
Hotel	720×576	414,720	7.546	4.861	4.854	4.732	4.987
Lena	512×512	262,144	7.348	4.374	4.467	4.342	3.849
Malamute	1616×1080	1,745,280	7.792	4.783	4.714	4.620	4.711
Man	1024×1024	1,048,576	7.524	5.058	5.084	4.936	5.711
Monarch	768×512	393,216	7.18	4.143	4.1442	4.095	3.887
Mushrooms	321×481	154,401	7.585	5.129	5.161	5.067	8.078
Parrots	768×512	393,216	7.256	3.945	3.988	3.828	2.884
Pens	512×480	245,760	7.482	4.368	4.268	4.188	3.393
Peppers	512×512	262,144	7.594	4.828	4.859	4.942	5.747
Rainier	1920×1080	2,073,600	7.088	4.499	4.466	4.298	7.699
Sun	2100×2034	4,271,400	6.950	3.295	3.577	2.736	1.274
Yachts	512×480	245,760	7.560	4.369	4.302	4.148	3.423
Zelda	720×576	414,720	7.334	4.265	4.127	4.112	3.226
Average				4.646	4.666	4.516	

^1^ Information entropy of the raw data. ^2^ Information entropy obtained by the multifunction local predictor (next-right). ^3^ Information entropy gained by the multifunction local predictor (next-bottom). ^4^ Information entropy achieved by the JPEG LS predictor.

**Table 4:** Compression achieved with different methods.

Image	JPEG LS		JPEG 2000		PNG		FLoCIC-IC		FLoCIC-AC	
	Size ^1^	bpp	Size ^1^	bpp	Size ^1^	bpp	Size ^1^	bpp	Size ^1^	bpp
Baboon	196,391	5.99	200,243	6.11	203,848	6.22	206,403	6.30	206,500	6.30
Balloon	149,322	2.88	157,296	3.03	177,426	3.42	170,141	3.28	162,094	3.13
Barbara	165,590	5.05	168,083	5.13	186,008	5.68	178,745	5.46	189,943	5.80
Barb2	241,027	4.65	248,400	4.79	266,756	5.15	265,877	5.13	269,352	5.20
Board	188,814	4.65	195,656	4.79	208,722	5.15	213,550	5.13	205,134	5.20
Boats	202,388	3.90	210,879	4.07	224,294	4.33	226,595	4.37	223,740	4.32
Cam.	38,137	4.66	40,850	4.99	42,403	5.18	41,142	5.02	43,310	5.29
Flower	106,946	3.48	108,459	3.53	124,291	4.05	120,787	3.93	119,130	3.88
Fruits	113,000	3.68	114,440	3.73	129,596	4.22	123,800	4.03	123,770	4.03
Girl	201,917	3.90	210,696	4.06	224,736	4.34	226,619	4.37	218,513	4.22
Gold	230,562	4.45	238,785	4.61	243,019	4.69	249,535	4.81	245,006	4.73
Hotel	225,316	4.35	237,861	4.59	248,916	4.80	248,076	4.79	246,013	4.75
Lena	130,704	4.00	134,417	4.10	145,634	4.44	143,259	4.37	142,789	4.36
Malamut	2,234,680	4.14	2,225,757	4.06	2,394,883	4.65	2,467,192	4.43	2,295,195	4.62
Man	611,909	4.67	632,163	4.82	650,599	4.96	658,921	5.03	649,298	4.95
Monarch	180,141	3.67	187,507	3.82	208,813	4.25	202,592	4.12	202,121	4.11
Mush.	84,815	4.40	87,818	4.55	98,581	5.11	91,161	4.72	98,716	5.12
Parrots	170,184	3.46	172,913	3.52	193,340	3.93	190,019	3.87	188,968	3.85
Pens	119,155	3.88	121,308	3.95	133,358	4.34	130,957	4.26	129,120	4.20
Peppers	146,630	4.48	151,739	4.63	160,465	4.90	166,822	5.01	162,562	4.96
Rainier	878,701	3.39	891,800	3.44	933,263	3.60	890,090	3.43	1,115,931	4.30
Sun	1,336,035	2.50	1,727,103	3.24	1,595,790	2.99	1,499,305	2.81	1,463,057	2.74
Yachts	115,183	3.75	120,225	3.91	132,198	4.30	126,798	4.13	127,960	4.17
Zelda	200,142	3.86	201,273	3.88	214,799	4.14	226,216	4.36	213,569	4.12
Average		4.03		4.18		4.49		4.43		4.46

^1^ Size is given in bytes.

## Data Availability

Not applicable.
